# The estimated influence of assumed physicians’ advice for tobacco smoking cessation among current smokers in Shanghai, China: A cross-sectional study

**DOI:** 10.18332/tid/153508

**Published:** 2022-10-17

**Authors:** Xiangjin Gao, Fanlingzi Shen, Siyuan Li, Rui Zhang, Wencheng Jiang, Bin Li, Ruiping Wang

**Affiliations:** Clinical Research Center, Shanghai Skin Diseases Hospital, Tongji University, Shanghai, China; School of Public Health, Shanghai University of Traditional Chinese Medicine, Shanghai, China

**Keywords:** physicians’ advice for quit, non-communicable diseases, smoking intensity, current smoker, smoking cessation intention

## Abstract

**INTRODUCTION:**

Evidence indicates that physicians’ smoking cessation advice is significant for tobacco control, which is an impetus to encourage smoking cessation among smokers, but the estimated influence of physicians’ smoking cessation advice on smokers’ intention to quit is limited in Shanghai, China.

**METHODS:**

We enrolled 1104 participants who were current smokers in the SJ (Songjiang) and FX (Fengxian) districts in Shanghai in 2021. An electronic questionnaire was used to collect data and SAS 9.4 was used for data analysis. Univariate and multivariate logistic regression was used to calculate odds ratios (ORs) with 95% confidence intervals (CIs) to estimate the influence of the assumed physicians’ advice for smoking cessation on current smokers’ smoking cessation plan.

**RESULTS:**

A total of 1104 participants provided information of which 914 were male smokers (82.8%) and 190 (17.2%) were female smokers. Multivariate logistic regression demonstrated that female smokers (OR=2.47; 95% CI: 1.66–3.68), smokers with at least 1 type of non-communicable disease (OR=2.09; 95% CI: 1.42–3.07), smoking intensity <20 cigarettes/day (OR=1.64; 95% CI: 1.22–2.17), with personal tobacco burden less than 20% (OR=1.52; 95% CI: 1.10–2.13), exposed to secondhand smoke (OR=1.99; 95% CI:1.44–2.76), and previous smoking cessation attempt (OR=4.43; 95% CI: 3.23–6.08), were more likely to report an intent to quit smoking. Moreover, approximately 50% of participants without a plan to quit in a year had also reported their intention to quit smoking with the presumption that the physicians would advise them to quit, irrespective of their sex, age, NCD status and secondhand tobacco smoke exposure.

**CONCLUSIONS:**

Physicians’ cessation advice could promote smokers to consider stopping smoking. The reported cessation intention was higher among female smokers, and smokers with NCD, lower smoking intensity and burden, with smoking cessation attempts, all of which could be incorporated into the implementation of tobacco control measures in the future in Shanghai.

## INTRODUCTION

Tobacco use is the leading preventable cause of premature death and diseases worldwide^1-3^. Each year, over 5 million people die due to tobacco-related diseases, and this figure is expected to increase to over 8 million by 2030 without any additional tobacco control efforts^4-7^. In order to respond to the globalization of the tobacco epidemic, the World Health Organization (WHO) Framework Convention on Tobacco Control (FCTC) was adopted by the 56th World Health Assembly in 2003 and became international law in 2005^8^. The WHO FCTC and its associated guidelines provide the foundation for tobacco control management and implementation^9^. In 2008, the WHO identified 6 evidence-based tobacco control measures to reduce tobacco use at the country level^10^. Known as MPOWER, they comprised best practice interventions and selected demand reduction measures outlined in the WHO FCTC to assist countries in the implementation of effective measures to reduce tobacco demand^11^.

To achieve the WHO targeted goal of reducing 30% tobacco consumption by 2025^12^, countries around the world implement tobacco control measures and policies that are outlined^13,14^ in the WHO FCTC, such as enacting regulations to protect the public from tobacco exposure, health literacy campaigns, advertisement restrictions, offering help to quit, graphic warning labels, and tobacco product tax increases etc. Among MPOWER measures, offering help to quit tobacco use (the O measure) is often highlighted as an effective measure^15^. The share of the world population covered by MPOWER measures at the highest level of achievement was 32% for O (cessation program offers help to quit) in 2018^16^. As one important component of the O measure of MPOWER, WHO proposes that healthcare professionals assume responsibility to help smokers quit^17^, and evidence has proven that physicians’ smoking cessation advice is important for tobacco control and an impetus to promote smoking cessation^18,19^. So, physicians’ advice plays an important role in curbing the high prevalence of tobacco smoking worldwide. Evidence indicates that the provision of smoking cessation service in clinical settings offers the opportunity for more efficient and effective cessation assistance^20^, and physicians’ cessation advice during medical consultations is an affordable intervention that increases the smoking cessation rate among smokers^21^. However, the availability of clinical setting consultation and physicians’ cessation services are extremely limited in China^22^. Studies demonstrate that fewer than 50% of physicians ask about patients’ tobacco use status, and only 30% of physicians think smokers will follow cessation advice they provide, and fewer than 7% of physicians provide tobacco cessation advice^22^. The possible barriers9 limiting smoking cessation services include: 1) lack of time, 2) lack of confidence in addressing smoking cessation, 3) lack of training in smoking cessation treatment, 4) outdated attitude toward tobacco addiction; and 5) absence of effective performance incentives etc. The insufficient smoking cessation services mainly contribute to the low tobacco cessation rate among smokers, so improving the utilization and availability of tobacco control is urgent to reach the under-served smoking population in China^23^.

China has the largest proportion of tobacco smokers, which consumes almost 2.5 trillion cigarettes per year and accounts for approximately 40% of global cigarette sales^24^. The high tobacco smoking prevalence among Chinese men contributed to 20% of all male adult deaths during the past decade. If the current situation continues, the number of smoking-related deaths each year will rise to 2 million by 2030 and 3 million by 2050^5,25,26^. However, smokers’ tobacco smoking histories are rarely inquired during visits to their physicians^26^. It seems that physicians neglect the duty to assist patients with smoking cessation as recommended by WHO^27^. China signed the WHO FCTC in 2005, but compliance with FCTC requirements has been slow in China^28,29^. In Shanghai, for example, the smoking rate in adults only decreased by 0.3% between 2017 and 2018^30^. In order to reduce smoking prevalence and the disease burden associated with tobacco, physicians are recommended to provide smoking cessation services to patients. Although systematic reviews indicate that physicians’ brief cessation advice increases the abstinence rate by about 48% in western countries^31^, there is still limited evidence regarding the estimated influence of physicians’ advice on smokers’ intention to quit in China^23,30-35^.

In this study, we implement a cross-sectional study among smokers in Shanghai to ascertain the characteristics of current smokers, their smoking cessation plan, and their responses to the assumed physicians’ advice for quitting, and to explore the influencing factors associated with smokers’ intention to quit due to assumed smoking cessation in Shanghai, China.

## METHODS

### Study population

This study was conducted in 2021 in the SJ (Songjiang) and FX (Fengxian) districts, which are rural areas in Shanghai. We investigated only two districts, due to our limited resources, and a multistage sampling design was applied for participant selection. First, we randomly selected the 2 districts (SJ and FX) out of the 15 districts in Shanghai. Second, 5 out 12 sub-districts in FX and 7 out of 15 sub-districts in SJ were randomly selected. Third, 2 residential communities were selected randomly in each of the 12 selected sub-districts. Fourth, a complete list of home addresses in each selected residential community was compiled previously, and 100 households were extracted randomly from the list without replacement. Fifth, 100 enumerated households were randomly ordered, and current smokers were then approached, following the randomized order, until 50 smokers were selected in each selected residential block^36^. Finally, a total of 1200 current smokers were proposed to be investigated. In this study, smokers aged >18 years of both sexes were included, and those who were unwilling to participate or with barriers in verbal communication were excluded. Finally, 1104 (92%) current smokers completed the questionnaire interview and were included in the final data analysis.

### Data collection

We applied an Android pad-assisted electronic questionnaire to collect data, and the whole audio recording process was convenient for subsequent data quality inspection. The split-half reliability coefficient of this questionnaire was 0.87 and a content validity coefficient of 0.85 was validated in a pilot study^36^. The questionnaire included four parts^36^: 1) demographic characteristics (age, sex, marriage status, education level, and vocation); 2) eight types of non-communicable diseases (NCDs); 3) tobacco use information (number of years as a smoker, daily tobacco consumption, cigarette retail price, previous smoking cessation history); and 4) two questions for tobacco smoking cessation intention (‘Do you have a plan to quit smoking in a year ?, and ‘Do you have a plan to stop smoking if a physician advised you to quit?, with response options, 1=Yes, 2=No).

### Definition and index calculation

We defined current smokers as those who smoked ≥100 cigarettes in their lifetime and were still smoking at the investigation time, and secondhand smoke exposure was defined for those who were exposed to tobacco smoke for >15 minutes per day for at least one day per week at home or the workplace. Smoking cessation intention due to physicians’ advice was defined for those who reported the intention to stop smoking if a physician advised them to quit (for the current smokers who answered ‘Yes’ to the question ‘Would you have a plan to stop smoking if a physician advised you to quit?’). The smoking duration was calculated as the time interval between the age of smoking initiation and the age at the questionnaire interview, and categorized as <10, 10–20, and >20 years. We define smoking intensity as daily consumed cigarettes and classified as <20 and ≥20 cigarettes/day. Tobacco burden (%) was calculated as the proportion of personal monthly tobacco consumption expense divided by personal monthly income and categorized as <20% and ≥20%^25^. Age was classified as <35, 35–44 and ≥45 years. Education level was categorized into three groups according to academic qualifications (junior high school or lower, senior high school, college or above). We classified individual monthly income as <5000, 5000–10000, and >10000 RMB (with 100 Chinese Renminbi about 14 US$).

### Data analysis

We employed SAS 9.4 software for data analysis. The quantitative data with normal distribution are given as mean with standard deviation (SD), and the quantitative data with skewed distribution are given as median with interquartile range (IQR). The qualitative data are presented as frequencies (n) and proportion (%). Student’s t-test and Mann-Whitney U test were applied to test the difference between two groups of quantitative data with normal or skewed distribution, respectively, and the chi-squared test was applied to test the difference between groups of qualitative data. We employed multivariable logistic regression (LR) to calculate odds ratios (OR) and 95% confidence intervals (CIs) to estimate the influence of the assumed physicians’ advice for quitting on current smokers’ smoking cessation intention and its associated influencing factors, covariables adjusted in LR were selected and ascertained based on the univariable logistic regression analysis for variables with p<0.05. Figures are given to show the exclusive impact of assumed physicians’ advice for quitting among smokers without intention to quit in a year, and the association between illness and intention to quit due to the assumed physicians’ advice for quitting among current smokers. A two-tailed p<0.05 was considered statistically significant.

## RESULTS

A total of 1104 smokers participated in the study; 914 male smokers (82.8%) and 190 female smokers (17.2%). The age of participants ranged 20–89 years, with a mean value of 43.6 years. Approximately 85% of participants were married, and 63% had an education level of college or above. Nearly half of the participants were professional workers, and the proportion of laborers and unemployed were 33% and 22%, respectively. Approximately one-quarter of current smokers had <5000 RMB personal monthly income, and about 22% of current smokers had one or more NCDs. Compared with male smokers, female smokers had a higher proportion of married status, college or above education level, and personal monthly income <5000 RMB, but a lower prevalence of NCD, all of these differences were statistically significant (p<0.05). Meanwhile, in comparison with smokers aged <35 years, smokers aged 35–44 or ≥45 years had higher married status proportion, junior high school or lower education level proportion, and NCD prevalence, all of the aforementioned differences were also statistically significant (p<0.05) (Table 1).

**Table 1:** The demographic characteristics of current smokers by age and gender, in rural areas of Shanghai, China (N=1104)

*Characteristics*	*Total smokers (n=1104) n (%)*	*Smokers by sex*		*p*	*Smokers by age (years)*			*p*
		*Male (n=914) n (%)*	*Female (n=190) n (%)*		*<35 (n=262) n (%)*	*35–44 (n=345) n (%)*	*≥45 (n=497) n (%)*	
**Marital status**				0.011				0.000
Married	938 (84.96)	788 (86.21)	150 (78.95)		176 (67.18)	304 (88.12)	458 (92.15)	
Unmarried/divorced/other	166 (15.04)	126 (13.79)	40 (21.05)		86 (32.82)	41 (11.88)	39 (7.85)	
**Education level**				0.011				0.000
Junior high school or lower	140 (12.68)	128 (14.00)	12 (6.32)		3 (1.15)	21 (6.09)	116 (23.34)	
Senior high school	272 (24.64)	226 (24.73)	46 (24.21)		46 (17.56)	62 (17.97)	164 (33.00)	
College or above	692 (62.68)	560 (61.27)	132 (69.47)		213 (81.30)	262 (75.94)	217 (43.66)	
**Occupation**				0.023				0.001
Laborers	359 (32.52)	304 (33.26)	55 (28.95)		96 (36.64)	108 (31.30)	155 (31.19)	
Professionals	502 (45.47)	423 (46.28)	79 (41.58)		81 (30.92)	162 (46.96)	259 (52.11)	
Unemployed/retired/others	243 (22.01)	187 (20.46)	56 (29.47)		85 (32.44)	75 (21.74)	83 (16.70)	
**Individual monthly income** (RMB)				0.004				0.000
<5000	279 (25.27)	215 (23.52)	64. (33.68)		86 (32.82)	67 (19.42)	126 (25.35)	
5000–10000	634 (57.43)	345 (37.75)	67 (35.26)		81 (30.92)	112 (32.46)	219 (44.06)	
>10000	191 (17.30)	354 (38.73)	59 (31.05)		95 (36.26)	166 (48.12)	152 (30.58)	
**Residency status**				0.353				0.000
Local resident	942 (85.33)	784 (85.78)	158 (83.16)		169 (64.50)	297 (86.09)	476 (95.77)	
Non-local resident	162 (14.67)	130 (14.22)	32 (16.84)		93 (35.50)	48 (13.91)	21 (4.23)	
**Non-communicabl edisease** (NCD)				0.000				0.000
								
At least 1 type	241 (21.83)	228 (24.95)	13 (6.84)		18 (6.87)	64 (18.55)	159 (31.99)	
0 type	863 (78.17)	686 (75.05)	177 (93.16)		244 (93.13)	281 (81.45)	338 (68.01)	

RMB: 100 Chinese Renminbi about 14 US$.

### Current smokers’ tobacco consumption condition

The median age for smoking initiation was 20 years, and 700 RMB for individual monthly tobacco expenses among current smokers; 59% of smokers had >20 years of tobacco smoking history, and approximately 40% of smokers had smoking intensity ≥20 cigarettes/day.

The prevalence of individual tobacco burden ≥20% among smokers was 23.9%. The prevalence of smoking cessation attempts and secondhand smoke exposure was 48% and 78%, respectively. Approximately 42% of current smokers reported planning to quit smoking in a year. In comparison with male smokers, female smokers had younger smoking initiation age (median: 18 years), a lower percentage of tobacco smoking years >20 years, and female smokers had more monthly expenses on tobacco consumption and higher personal tobacco burden; they also had a lower proportion of previous smoking cessation attempt and secondhand smoke exposure, all of these differences were statistically significant (p<0.05). Meanwhile, in comparison with smokers aged <35 years, smokers aged 35–44 years and ≥45 years had a higher percentage of tobacco smoking year over 20 years, and a higher proportion of smoking intensity ≥20 cigarettes/day; and elder smokers also tended to had a higher proportion of smoking cessation intention in a year, all of these differences were statistically significant (p<0.05) (Table 2).

**Table 2:** The smoking intensity, smoking duration, tobacco expenses, and smoking cessation attempts among current smokers by gender and age, in the rural area of Shanghai, China (N=1104)

*Variables*	*Total smokers (n=1104) n (%)*	*Smokers by sex*		*p*	*Smokers by age (years)*			*p*
		*Male (n=914) n (%)*	*Female (n=190) n (%)*		*<35 (n=262) n (%)*	*35–44 (n=345) n (%)*	*≥45 (n=497) n (%)*	
**Smoking initiation age** (years), median (IQR)	20 (18–23)	20 (18–25)	18 (18–19)	0.000	19 (18–20)	19 (18–22)	20 (18–25)	0.000
**Tobacco smoking years**				0.024				0.000
<10	166 (15.04)	132 (14.44)	34 (17.89)		127 (48.47)	39 (11.30)	0 (0.00)	
10–20	288 (26.09)	227 (24.84)	61 (32.11)		135 (51.53)	122 (35.36)	31 (6.24)	
>20	650 (58.88)	555 (60.72)	95 (50.00)		0 (0.00)	184 (53.33)	466 (93.76)	
**Smoking intensity** (cigarettes/day)				0.875				0.000
<20	668 (60.51)	554 (60.61)	114 (60.00)		182 (69.47)	230 (66.67)	256 (51.51)	
≥20	436 (39.49)	360 (39.39)	76 (40.00)		80 (30.53)	115 (33.33)	241 (48.49)	
**Monthly tobacco expense** (RMB), median (IQR)	700 (300–1000)	600 (300–1000)	1000 (1000–1000)	0.000	800 (400–1000)	800 (400–1000)	600 (300–1000)	0.219
**Personal tobacco burden** (%)				0.000				0.916
<20	840 (76.09)	734 (80.31)	106 (55.79)		194 (74.05)	272 (78.84)	374 (75.25)	
≥20	264 (23.91)	180 (19.69)	84 (44.21)		68 (25.95)	73 (21.16)	123 (24.75)	
**Previous smoking cessation attempt**				0.000				0.435
Yes	529 (47.92)	499 (54.60)	30 (15.79)		135 (51.53)	136 (39.42)	258 (51.91)	
No	575 (52.08)	415 (45.40)	160 (84.21)		127 (48.47)	209 (60.58)	239 (48.09)	
**Secondhand smoke exposure**				0.000				0.042
Yes	857 (77.63)	733 (80.20)	124 (65.26)		199 (75.95)	254 (73.62)	404 (81.29)	
No	247 (22.37)	181 (19.80)	66 (34.74)		63 (24.05)	91 (26.38)	93 (18.71)	
**Plan to quit smoking in a year**				0.000				0.043
Yes	462 (41.85)	414 (45.30)	48 (25.26)		106 (40.46)	125 (36.23)	231 (46.48)	
No	642 (58.15)	500 (57.70)	142 (74.74)		156 (59.54)	220 (63.77)	266 (53.52)	

IQR: interquartile range. RMB: 100 Chinese Renminbi about 14 US$.

### Smoker’ response to the assumed physicians’ advice to quit

**Figure 1:**
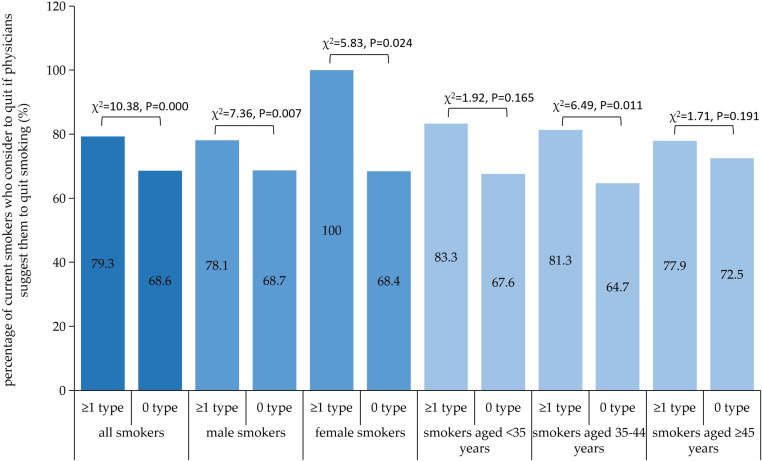
The percentage of current smokers (total, divided by sex or age) who will consider quitting tobacco smoking if physicians provide a suggestion to quit for them both among those with at least 1 type of NCD (noncommunicable disease) or without NCD

In all, 70.92% (783/1104) of smokers reported their intention to quit smoking if physicians advised them to quit. Male smokers had a slightly higher percentage of smoking cessation intention than female smokers due to physicians’ advice, but without statistical significance. Univariate logistic regression (LR) showed that smokers aged ≥45 years, with non-communicable diseases (NCDs), with smoking intensity <20 cigarettes/day, with personal tobacco burden <20%, with secondhand tobacco smoke exposure and with previous smoking cessation attempts, had higher smoking cessation intention due to the assumed physicians’ advice to quit. Figure 1 indicates that smokers with NCD had a higher intention to quit due to the assumed physicians’ advice than those without NCD, both among female and male smokers (p<0.05). The multivariate LR analysis indicated that female smokers (OR=2.47; 95% CI: 1.66–3.68), smokers with NCD (OR=2.09; 95% CI: 1.42–3.07), smokers with smoking intensity <20 cigarettes/day (OR=1.64; 95% CI: 1.22–2.17), with tobacco burden <20% (OR=1.52; 95% CI: 1.10–2.13), with secondhand smoke exposure (OR=1.99; 95% CI: 1.44–2.76), and with previous smoking cessation attempt (OR=4.43; 95% CI: 3.23–6.08), had a higher smoking cessation intention due to the assumed physicians’ advice to quit (Table 3).

**Table 3:** The likelihood of estimated smoking cessation intention due to assumed physicians’ advice for quitting among current smokers, in a rural area of Shanghai, China

*Variables*	*Smoking cessation intention due to assumed physicians’ advice n (%)*	*Model A*		*Model B ^a^*	
		*OR*	*95% CI*	*AOR*	*95% CI*
**Sex**					
Male (Ref.)	649 (71.01)	1		1	
Female	134 (70.53)	0.98	0.69–1.38	**2.47**	**1.66–3.68**
**Age** (years)^b^					
<35 (Ref.)	180 (68.70)	1		1	
35–44	234 (67.83)	0.96	0.68–1.36	1.15	0.78–1.67
≥45	369 (74.25)	**1.31**	**1.00–1.83**	1.34	0.93–1.94
**Marital status**					
Married	657 (70.04)	0.74	0.51–1.08		
Unmarried/divorced/other (Ref.)	126 (75.90)	1			
**Education level**					
Junior high school or lower (Ref.)	106 (75.71)	1			
Senior high school	193 (70.96)	0.78	0.49–1.25		
College or above	484 (69.94)	0.75	0.49–1.14		
**Individual monthly income** (RMB)					
<5000 (Ref.)	195 (69.89)	1			
5000–10000	279 (67.72)	0.90	0.65–1.26		
>10000	309 (74.82)	1.28	0.91–1.80		
**Non-communicable disease^b^**					
At least 1 type	191 (79.25)	**1.75**	**1.24–2.46**	**2.09**	**1.42–3.07**
0 type (Ref.)	592 (68.60)	1		1	
**Smoking intensity** (cigarettes/day)^b^					
<20	495 (74.10)	**1.47**	**1.12–1.92**	**1.64**	**1.22–2.17**
≥20 (Ref.)	288 (66.06)	1		1	
**Smoking duration** (years)					
<10 (Ref.)	114 (68.67)	1			
10–20	210 (72.92)	1.29	0.81–1.87		
>20	459 (70.62)	1.10	0.76–1.59		
**Personal tobacco burden** (%)^b^					
<20	622 (74.05)	**1.85**	**1.37–2.44**	**1.52**	**1.10–2.13**
≥20 (Ref.)	161 (60.98)	1		1	
**Secondhand smoke exposure^b^**					
Yes	635 (74.10)	**1.91**	**1.42–2.58**	**1.99**	**1.44–2.76**
No (Ref.)	148 (59.92)	1		1	
**Previous smoking cessation attempt^b^**					
Yes	448 (84.69)	**3.96**	**2.97–5.29**	**4.43**	**3.23–6.08**
No (Ref.)	335 (58.26)	1		1	

Model A: univariate logistic regression. Model B: multivariate logistic regression.

a The co-variables adjusted in model B were ascertained based on model A of which variables with a p<0.05 were selected and sex variable as well.

b The difference between groups was statistically significant (p<0.05). AOR: adjusted odds ratio. The bold values indicate statistically significant (p<0.05). RMB: 100 Chinese Renminbi about 14 US$.

### The exclusive influence of the assumed physicians’ advice to quit

**Figure 2:**
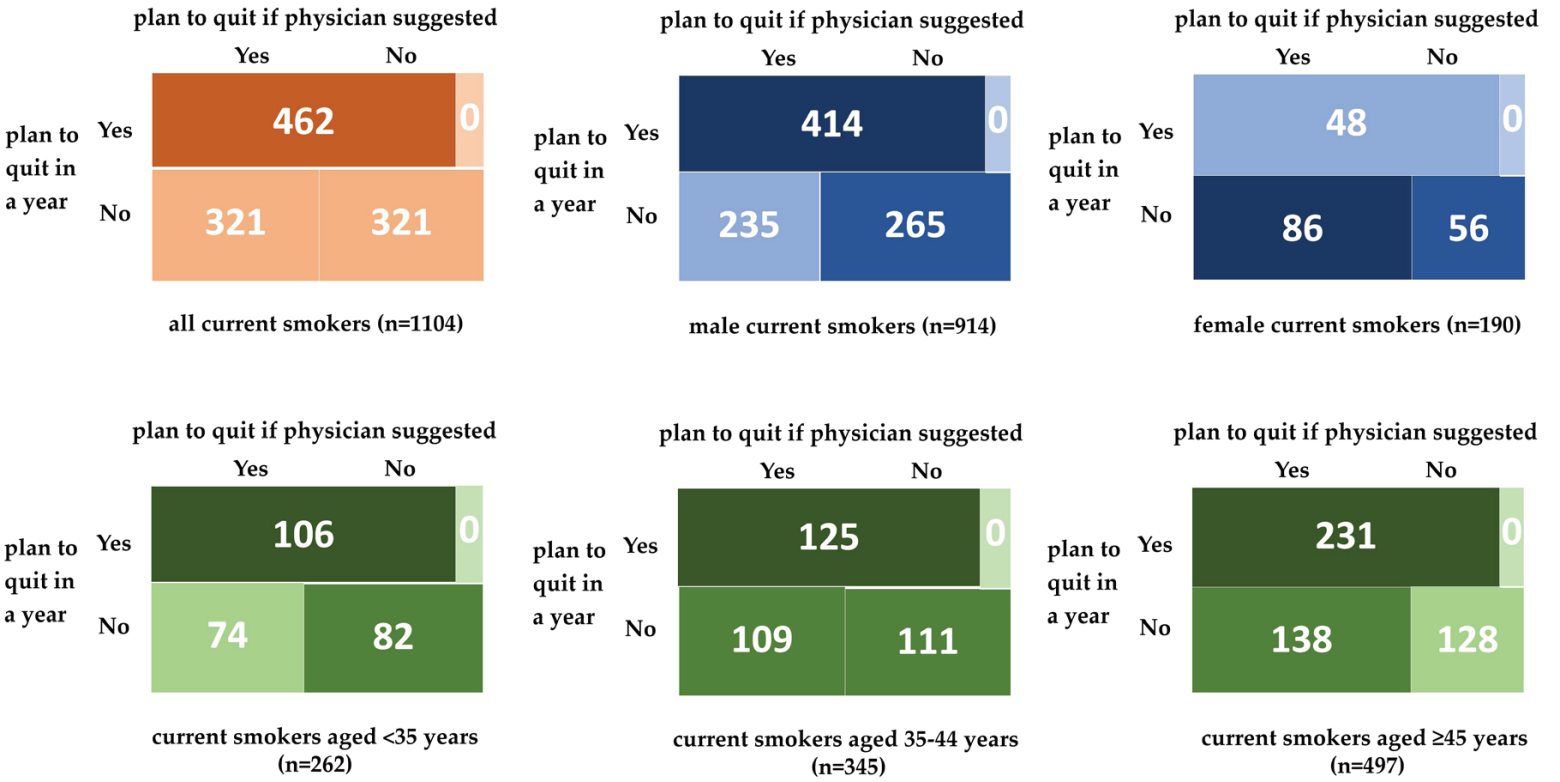
The number of current smokers (total, divided by sex or age) who will consider quitting tobacco smoking if physicians provide a suggestion to quit for them both among those who plan to quit in a year or not

**Figure 3:**
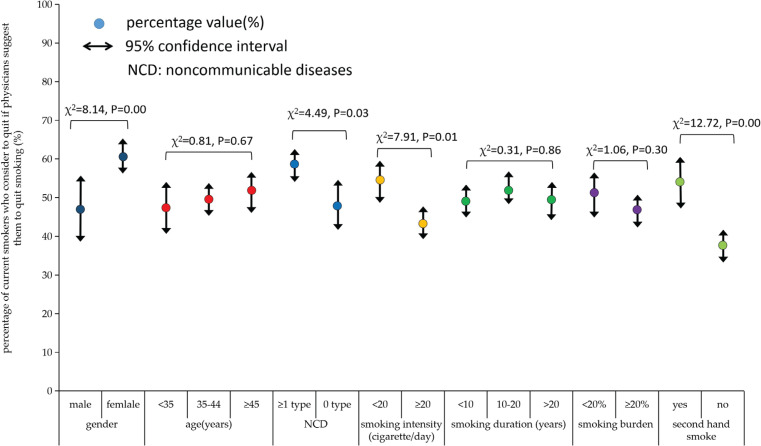
The percentage of current smokers with different features who will consider quitting tobacco smoking if physicians provide suggestions to quit for them among those who have no plan to quit in a year (n=642)

In order to evaluate the exclusive influence of the assumed physicians’ advice on smokers’ intention to quit, we divided the 1104 smokers into group A (n=462, smokers with a plan to quit in a year) and group B (n=642, smokers without a plan to quit in a year.). Figure 2 indicates that 642 out of 1104 current smokers reported no plan to quit in a year (58.15%), but half would consider stopping smoking if their physician would advise them to quit. Subgroup analysis showed that 47% of male smokers, 61% of female smokers, and 47–52% of smokers in three age groups would consider stopping smoking if a physician advised them to quit among those without a plan to quit in a year. Moreover, among the 642 smokers in group B, female smokers reported higher intention to quit if physicians advised them to quit; and smokers with NCD, with tobacco smoking intensity <20 cigarettes/day, with secondhand smoke exposure, had higher smoking cessation intention due to the assumed physicians’ advice to quit (Figures 2 and 3).

## DISCUSSION

Smoking cessation is the most effective measure for smoke-related diseases and death prevention^37^, and quitting smoking can lead to substantial health gains, even later in life^38^. In this study, we discovered that 42% of current smokers in Shanghai reported the intention to quit in a year, and approximately 70% would consider quitting smoking if physicians advised them to quit. Moreover, nearly half of current smokers without a plan to quit in a year had reported the intention to quit due to the assumed physicians’ advice to quit. The finding in this study was in line with previous studies^5,22^. A robust body of literature has demonstrated that brief smoking cessation advice from a healthcare provider during usual medical consultation could encourage smoking cessation^9,20,21,39^. A meta-analysis showed that the smoking cessation rate among smokers who followed physicians’ advice was apparently higher than those without physicians’ advice^34^. A randomized control trial showed that physicians’ advice for tobacco control intervention was a valid way to promote smoking cessation among smokers^35^. So, physicians’ advice for quitting as an important part of the O measure of MPOWER, is an effective measure for tobacco control among smokers.

Previous studies have demonstrated that smokers who smoked less each day were more likely to quit smoking^40^. We noticed that smokers who smoked <20 cigarettes/day reported higher smoking cessation intention due to the assumed physicians’ advice to quit, which was in line with previous findings. Moreover, this study indicates that smokers with higher smoking intensity and heavier personal tobacco burden had lower smoking cessation intention, even with the assumed physicians’ advice for quitting. The lower smoking cessation intention among smokers might indicate heavier nicotine dependence and lower awareness of smoking cessation benefits. Thus, the health office should implement more health education and health promotion activities to advocate the harm of tobacco smoking and the benefit of smoking cessation among smokers in the future.

This study demonstrated that smokers with NCD were more sensitive to the assumed smoking cessation advice from physicians, these findings are consistent in the subgroup analysis (both in male and female smokers, and in smokers of all ages), this might be due to the fact that smokers with NDC were worried about their disease progression. A previous study showed that illness can be a powerful motivation to quit among smokers^9^. So, in order to curb the high smoking prevalence and promote smoking cessation, it is crucial to pay special attention to smokers with NCD, provide professional smoking cessation counselling, psychological support and individualized treatment based in the clinical setting, which can effectively improve the success of smoking cessation among current smokers. Moreover, we also noticed that smokers of older age were prone to report higher smoking cessation intention with the assumed quit advice of physicians. In China, older age predicts longer smoking duration and lower economic level, and older smokers are more susceptible to many senile diseases, which might make them more receptive to smoking cessation advice from physicians.

Most adults who smoke wish to quit, but quitting smoking is extremely challenging^38^, and the quit rate was low among Chinese smokers which was 14.4% in 2015^41^. In this study, nearly 50% of current smokers had previous smoking cessation attempts but all relapsed, which might be mainly due to their unassisted smoking cessation attempts. Previous studies indicate that the abstinence rate for unaided smoking cessation is typically lower than 5%, and even simple advice or basic clinical intervention from physicians can increase the abstinence^42^. In this study, current smokers with previous cessation attempts reported 4.43 times higher smoking cessation intention due to the assumed physicians’ advice than those without previous cessation attempt experience. This indicates that the assisted cessation service from physicians and other health professionals could increase the quit rate among smokers, and providing individualized counselling can effectively assist smokers to stop smoking^23^.

In contrast to the western world, workplace smoking in China remains common and is a major source of secondhand smoke exposure. An investigation implemented in 2018 indicated that 51% of adults were exposed to tobacco smoke on the job^43^. In this study, approximately 78% of smokers reported secondhand smoke exposure at home or in the workplace, this indicated that smoking remains highly normalized in China, and promoting smoking cessation is crucial and urgent among Chinese smokers. Interestingly, in this study, with the assumed physicians’ advice to quit, smokers with secondhand smoke exposure reported higher intention to quit than those without. This might due to the fact that smokers who reported secondhand smoke exposure were mainly light smokers and female smokers, they were prone to have a higher awareness of tobacco harm, and responded more positively to physicians’ advice for quitting.

Although the proportion of smokers receiving smoking cessation assistance is low in China, medical practitioners are still playing an increasingly important role in health education and promotion, and they have an increasing array of options to assist with smoking cessation services for smokers^44^. Previous studies demonstrated that cessation advice of medical practitioners is significant in reducing the prevalence of tobacco smoking, and the exclusive influence could be as large as the number of the general population that frequently seek medical consultations^34^. As previously discussed, healthcare providers have a good opportunity to provide patients with smoking cessation advice during their hospital visits. Generally, smoking cessation advice from healthcare providers was usually provided to smokers who have the intention of smoking cessation. However, it would be more beneficial for medical practitioners to provide smoking cessation aid to the whole smoking population, not just to those who plan to quit smoking^21^. Therefore, we recommend that the health office should consider incorporating smoking cessation advice of medical practitioners into relevant laws and regulations and spending more efforts to train physicians to provide brief smoking cessation interventions more effectively in the future. Moreover, we should note that the high proportion of intention to quit did not correspond to an actual high proportion of smoking cessation behavior, so the incorporation of measures to transfer intention to quit into realistic smoking cessation is important in future actions against the high tobacco smoking epidemic in China.

### Limitations

This study has several limitations. First, the selected current smokers in this study did not constitute a random sample of the total present smoking population in Shanghai, which would limit the generalization of the findings. Second, questions in this study concerning the smoking cessation intention in a year and the intention to quit due to the assumed physicians’ advice to quit were just the attitude of current smokers during the questionnaire interviews but not their realistic behavior transformation, so the implementation of physicians’ tobacco control measure in actual clinical settings in the future would provide a chance to observe and estimate the real effect of physicians’ smoking cessation advice as a feasible measure to decrease smoking in Shanghai, China. Third, we only employed the smoking cessation advice of physicians to estimate its association with smokers’ intention to quit smoking. Other measures, such as health literacy campaigns, advertisement restrictions, graphic warning labels and tobacco product tax increases, might also influence the intention to quit. Fourth, information collected in this study was provided by smokers, which possibly leads to some recall bias. Therefore, incorporating some improvements to overcome these limitations, should be considered in future studies.

## CONCLUSIONS

The assumed physicians’ cessation advice could promote smokers to consider stopping smoking. The reported cessation intention was higher among female smokers, and smokers with NCD, and lower smoking intensity and burden, with smoking cessation attempts, all of which could be incorporated into the implementation of future tobacco control measures in Shanghai.

## Data Availability

The data supporting this research are available from the authors on reasonable request.
